# Variation in movement strategies: Capital versus income migration

**DOI:** 10.1111/1365-2656.13800

**Published:** 2022-09-05

**Authors:** Simon R. Evans, Stuart Bearhop

**Affiliations:** ^1^ Centre for Ecology and Conservation University of Exeter Penryn UK

**Keywords:** animal migration, migratory energetics, movement ecology, physiological ecology, stopover ecology

## Abstract

Abstract

1. Animal migrations represent the regular movements of trillions of individuals. The scale of these movements has inspired human intrigue for millennia and has been intensively studied by biologists.
2. This research has highlighted the diversity of migratory strategies seen across and within migratory taxa: while some migrants temporarily express phenotypes dedicated to travel, others show little or no phenotypic flexibility in association with migration. However, a vocabulary for describing these contrasting solutions to the performance trade‐offs inherent to the highly dynamic lifestyle of migrants (and strategies intermediate between these two extremes) is currently missing.
3. We propose a taxon‐independent organising framework based on energetics, distinguishing between migrants that forage as they travel (income migrants) and those that fuel migration using energy acquired before departure (capital migrants).
4. Not only does our capital:income continuum of migratory energetics account for the variable extent of phenotypic flexibility within and across migrant populations, but it also aligns with theoreticians' treatment of migration and clarifies how migration impacts other phases of the life cycle. As such, it provides a unifying scale and common vacabulary for comparing the migratory strategies of divergent taxa.

## Graphical abstract

A proposal for classifying migrations based on how they are fuelled, ranging from income migrants that feed as they move, to capital migrants that rely entirely on energy stores to complete their journey. This organising framework is taxon‐independent, facilitating comparisons across distantly related migratory taxa.

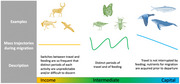

## 1 INTRODUCTION

Migration has independently evolved in all major vertebrate classes and is also well represented among insects and other invertebrates (Dingle, 2014). Accordingly, migrants are notable in both their taxonomic diversity and the range of behaviours that have evolved to facilitate this highly dynamic lifestyle. However, despite a huge body of work in this field, our ability to form a broad‐reaching synthesis of migration is hindered by the lack of a generalisable framework for organising behavioural variation in migratory strategies that is applicable to the full gamut of migrant taxa. Our understanding of the biology of migration has instead tended to progress along taxon‐specific lines of enquiry, such that even a broadly agreed definition of migration has proven elusive (Dingle, 2006; Kennedy, 1985; Milner‐Gulland et al., 2011; Newton, 2012; Taylor, 1986). Here, we take migration to be a repeated seasonal movement between discrete areas of residency (Berger, 2004; Newton, 2010; Soriano‐Redondo et al., 2020). This excludes cyclical movements on much shorter time‐scales (e.g. diel vertical migration) and less spatially or temporally predictable movements (e.g. nomadism, irruptive migration). However, we do not restrict this definition to the movement of *individuals*, as do many vertebrate ecologists, because while the short adult life span of many insects means that repeat movements on a seasonal time‐scale are unfeasible at the individual level, the same outcome at the genetic level is achieved via a multi‐generational movement cycle, with each generation dedicated to a single migratory journey, or a portion of it. We believe that if definitional arguments are put aside then the parallels that exist despite the different migratory units (i.e. individuals vs. lineages) can be instructive for understanding the evolution of this remarkable lifestyle. However, the greater emphasis on economic impact (Wotton et al., 2019) and the greater challenge of re‐sighting marked individuals (McCord & Davis, 2010) has meant that insect migration has focussed on population‐level phenomena, rather than the level of the individual at which phenotypic trade‐offs that influence fitness (and thus evolution) are typically manifested and resolved (though see Taylor et al., 2020). The taxonomic representation of the examples we cite is a necessary reflection of this.

In recent years, researchers have classified migrations according to their purpose, typically into two or three categories (Berdahl et al., 2016; Gnanadesikan et al., 2017; Shaw, 2016). Berdahl et al. (2016), for example, identify two distinct forms of migration. The first, ‘feeding migration’ [referred to as ‘tracking migration’ by Shaw (2016)], involves individuals following a looped route as they track favourable foraging conditions, as is common in marine fish and terrestrial mammals (Bohrer et al., 2014; Torney et al., 2018). Berdahl et al.'s (2016) second form of migration is ‘breeding migration’, in which individuals make to‐and‐fro movements between specific end‐points for the purposes of reproduction. Shaw (2016) recognises a third category, ‘refuge migration’, in which individuals leave their site of breeding to avoid harsh conditions that prevail for part of the year, distinguishing this from breeding migration on the basis that for breeding migrants the principal locations of feeding and breeding are spatially distinct. However, some species qualify as both breeding migrants and refuge migrants under this system (Gnanadesikan et al., 2017), and herring (*Clupea harengus*), which follow a fasted, circular migration between feeding, overwintering and spawning sites (Varpe et al., 2005), exhibit movement behaviour consistent with all three categories. Thus, although such classification systems have proved valuable in highlighting common factors favouring the emergence of migration, it is often difficult to identify a single purpose of migration.

Inevitably, given the demands of large‐scale movement, energetics is a key driver for the evolution of migration (Avgar et al., 2014; Hein et al., 2012; Piersma et al., 2022; Roff, 1991). Indeed, based on their fuelling strategies, Theunis Piersma organised migrating birds into a sequential – rather than strictly categorical – system. Piersma (1987) recognised ‘hop, skip and jump’ strategies among avian migrants, ranging from frequent stops (‘hop’ migration) through to ‘jump’ strategies in which large distances are covered between stopovers and refuelling periods are correspondingly extended. Bischof et al. (2012) propose that a strategic continuum is found among populations of grazing ungulates pursuing the highly nutritious spring growth of forage plants as it advances across the terrestrial landscape (i.e. along a vegetative phenological gradient). Some animals migrate in synchrony with the ‘green wave’ (the emergence of highly palatable, new‐growth leaves: Drent et al., 1978; Sawyer & Kauffman, 2011), effectively allowing them to prolong the springtime they experience at the most nutrition favourable point. The migration of surf scoter (*Melanitta perspicillata*) ducks provides a non‐herbivorous variant of this idea, tracking a ‘silver wave’ of Pacific herring (*Clupea pallasi*) spawning events northwards along the American Pacific coast (Lok et al., 2012). At the other extreme of Bischof et al.'s (2012) continuum are animals that remain at their wintering grounds as forage quality declines, before then migrating rapidly and without feeding interruptions, such that they overtake the green wave and effectively experience a second green‐up at their summer feeding grounds. Intermediate between these extremes of, on the one hand, ‘surfing’ the green wave and, on the other hand, jumping over it, Bischof et al. (2012) recognise a range of ‘hopping’ strategies, which clarifies the parallels with Piersma's (1987) stratification of shorebird migration strategies.

We propose that a migratory energetics continuum can be applied to all migrants and that such a perspective facilitates comparison of migratory strategies at multiple hierarchical levels – not only between species but also among and within conspecific populations, and even changes at the individual level. The capital:income framework we apply shares the central focus on energetics of Piersma (1987) and Bischof et al. (2012), but (i) is explicit in focussing on how migration is fuelled, rather than the distance travelled (cf. Piersma et al., 2005) (ii) can be applied to a broad diversity of migratory taxa, and (iii) expands the range of strategies. Indeed, it is motivated by a conviction that ‘long‐haul’ migrants (those that forego foraging and other activities as they travel) exhibit a distinct migratory syndrome to ‘stop‐start’ migrants, (a dichotomy previously noted by Blem, 1980). In particular, while some migratory species undergo conspicuous and extensive phenotypic reorganisation in association with migration, others appear to make minimal phenotypic adjustments across the migratory cycle, and we suggest this corresponds to their position on a continuum of migratory energetics.

**FIGURE 1:**
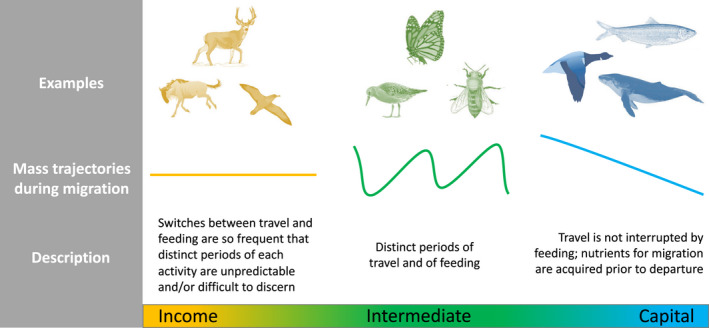
Summary of the three migratory energetic strategies, with exemplar species depicted as vignettes. Income migrants: mule deer (*Odocoileus hemionus*; Sawyer & Kauffman, 2011), blue wildebeest (*Connochaetes taurinus*; Torney et al., 2018), Cory's shearwater (*Calonectris diomedea*; Dias et al., 2012); intermediate strategy migrants: monarch butterfly (*Danaus plexippus*; McCord & Davis, 2010), red knot (*Calidris canutus*; Piersma et al., 2021), giant honeybee (*Apis dorsata*; Robinson, 2012); capital migrants: herring (*Clupea harengus*; Varpe et al., 2005), light‐bellied brent goose (*Branta bernicla*; Vissing et al., 2020), humpback whale (*Megaptera novaeangliae*; Whitehead & Moore, 1982).

At one end of our migratory energetics continuum are animals that forage throughout the migration cycle, including during the periods of travel. These individuals store minimal additional energy in advance of migration, relying instead on sourcing nutrients from the environments they are traversing as needed (Figure 1). At the other end of the continuum are migrants that complete their entire migratory journey without feeding between departure and arrival. Clearly, this latter strategy demands sufficient endogenous energy stores to fuel both migration and other bodily functions, and may involve considerable physiological reorganisation to accommodate the distinct demands of the various phases of the migratory cycle. We characterise these opposing strategies as *income migration* and *capital migration*, respectively, following analogous description of variation in the way that reproduction is energetically financed (Drent & Daan, 1980; Jönsson, 1997; Thomas, 1988). In truth, this is a continuum, with many populations adopting an intermediate migratory strategy by stopping periodically to fuel en route. However, this is also true of breeding strategies (Williams et al., 2017), for which the capital:income perspective has nonetheless proven enduringly useful (Davis et al., 2016; Whiteman et al., 2021). The now‐dominant interpretation understands capital breeding as being fuelled using resources acquired before breeding, while income breeders obtain the resources necessary for reproduction through concurrent foraging (Thomas, 1988). We argue that application of an equivalent energetics perspective to migration offers a taxon‐independent interpretation of variation in the behavioural changes associated with migration, thereby highlighting commonalities or explaining differences between migratory populations. For example, spring migration of various species of migratory deer is delayed by supplementary feeding in winter (Jones et al., 2014; Lewis & Rongstad, 1992; Peterson & Messmer, 2007), whereas for a long‐distance migratory bird (American redstart, *Setophaga ruticilla*) a delay in migration was instead seen in response to reduced food availability (Cooper et al., 2015). These contrary observations make sense if we consider the differing energetic strategies (Table 1): as income migrants, deer seek to secure food intake throughout migration, while capital migrants seek to reach a threshold energetic reserve that will allow them to complete their planned journey without interruption. [Correction added on 28 September 2022 after first online publication: the sentence For example, … is revised.]

**TABLE 1:** Summary of the typical differences between capital, intermediate and income migration

Classification	Income migration	Intermediate strategy migration	Capital migration
Description	Opportunistic feeding frequently interrupts travel	Stopovers for refuelling	Nonstop migration
Diagnostic feature	Mass stable	Migration divided into two or more periods of fasted travel, punctuated by foraging interruptions	Mass declines continuously throughout migration due to consumption of energy stores
Typical travel mode	All, but especially terrestrial	Flying, swimming	Flying, swimming
Phenotypic flexibility	Typically limited, although can be considerable in some aerial migrants, e.g. hoary bats (McGuire et al., 2013)	Variable	Extensive: coordinated reorganisation of morphological, physiological and behavioural phenotypes
Pre‐departure behavioural plasticity	Negligible, although modest increases in fat stores are possible	High: obligate hyperphagy	High: obligate hyperphagy
Adjustability of departure schedule	High	Medium: as for capital migrants, intermediate strategy migrants will undertake fasted journeys and likely rely on sites offering high but short‐lived resource availability	Low: morphological specialisation is a commitment to migration. Additionally, conditions on the arrival site may be stronger determinants of survival or reproductive success, and may be unrelated to conditions at the departure site
Trigger for departure	Departure occurs when conditions at nearby sites are superior to the current location	Departure dependent on endogenous energy stores	Departure dependent on endogenous energy stores
Time between origin and destination	Short	Long	Short
Post‐departure adjustability of travel schedule	Low: migration insensitive to resource availability along the route	Moderate: opportunistic interruptions for foraging are possible	Low: migration insensitive to resource availability along the route
Cost of foraging during migration	Very high: deviation from straight‐line course; cost of reversing phenotypic specialisation; greatly extends duration of migration	High but stopovers for feeding are essential	Very high: deviation from straight‐line course; cost of reversing phenotypic specialisation; greatly extends duration of migration

## 2 INCOME MIGRATION

Many terrestrial migrations are income‐based, with migrants foraging frequently throughout their journey, effectively adopting a ‘march‐and‐browse’ strategy. The mass migrations of ungulates, for example, are driven by seasonal changes in food quantity and quality (Fryxell et al., 1988): early in the growing season fresh growth is in limited supply but as that year's new growth matures its digestibility declines (Wilmshurst et al., 1995). This temporal trade‐off between forage quantity and forage quality drives the movement of herbivores as they seek to prolong access to forage at an intermediate stage of phenology (Albon & Langvatn, 1992). For this reason, ungulate migrants are often characterised as ‘green wave surfers’, with the physical progress of their migration across the landscape keeping pace with plant phenology (Bischof et al., 2012; Hebblewhite et al., 2008). Mule deer (*Odocoileus hemionus*), for example, make prolonged interruptions to the progress of their spring migration every 5 km, on average, to graze (Sawyer & Kauffman, 2011). Given this frequent switching between feeding and locomotion, we do not expect to see marked reorganisation of the phenotype specifically for migration apart from the behavioural changes that trigger, maintain and terminate the migratory journey itself. Indeed, it has been suggested that the activity level of migratory herbivorous mammals differs little across the migratory cycle (in contrast to, e.g. avian and insect migrants; Avgar et al., 2014), perhaps because the dispersed nature of forage prohibits sedentariness. Combined with the relatively gentle phenological gradient that drives their seasonal movement, this means that migration can be achieved simply by aligning small‐scale movements in a consistent direction, without much need for change to time budgets for locomotion versus feeding. Similar phenological gradient‐driven migrations have been proposed for long‐nosed bats (*Leptonycteris* spp.) tracking nectar‐pollen production of agaves (Fleming et al., 1993; Moreno‐Valdez et al., 2000) and for these we also expect migration‐associated phenotypic flexibility to be minimal because there is, again, little change in time dedicated to locomotion versus foraging during the migratory cycle. We also expect the migration schedule of income migrants to be flexible, since the phenology of their preferred food sources can be continually monitored and migration accelerated, slowed or even reversed as required (Bartlam‐Brooks et al., 2013).

A migratory strategy termed ‘fly‐and‐forage’ has been suggested to exist for some birds that hunt on the wing, since their foraging behaviours facilitate rapid switches between migration and foraging. Both hawks and owls have been observed carrying prey during migration (Kerlinger, 1989; Shelley & Benz, 1985), and it has been suggested that the atypical trans‐oceanic migrations of the insectivorous Amur falcon (*Falco amurensis*), which coincide with those of dragonflies, are timed to facilitate in‐flight foraging (Anderson, 2009). Strandberg and Alerstam (2007) report that a large majority (78%) of ospreys (*Pandion haliaetus*) migrating past a lake in Sweden exhibited fly‐and‐forage behaviour. Of course, such a strategy is only possible where compatible habitat is frequently traversed; when southbound ospreys reach the Sahara Desert the in‐flight time they devote to foraging is cut by 75% (Klaassen et al., 2008), demonstrating strategic flexibility. A similar shift in daily time budgets is shown by hobbies (*Falco Subbuteo*) and Eleonora's falcon (*F. eleanorae*) crossing the Sahara Desert on migration from Europe (Hadjikyriakou et al., 2020; López‐López et al., 2010; Strandberg et al., 2009).

Some seabirds also merge foraging activities with migration and can likewise be considered income migrants. Animal‐borne GPS tags show that Cory's shearwaters (*Calonectris diomedea*) follow a fly‐and‐forage strategy during their 13,000 km pelagic migration, with little evidence of increased fat stores immediately prior to departure (Dias et al., 2012). Seabird activities during migration remain relatively poorly studied so it is currently difficult to know whether the income migration strategy is widespread but it is certainly not the case that all seabirds are as dedicated to income migration as Cory's shearwaters. Their close relative, the sooty shearwater (*Puffinus griseus*), for example, also forages during migration (Spear & Ainley, 1999) but individuals increase their fat stores prior to departure and spend more time in flight during the migration period (Hedd et al., 2012) than do Cory's shearwaters. Conversely, other seabird species (Manx shearwater, *Puffinus puffinus*: Guilford et al., 2009; Arctic terns, *Sterna paradisaea*: Egevang et al., 2010; South Polar skuas, *Catharacta maccormicki*: Kopp et al., 2011; long‐tailed skuas, *Stercorarius longocaudus*: Sittler et al., 2011) stop more frequently than Cory's shearwaters, suggesting they also rely on mid‐journey foraging to fuel migration.

## 3 CAPITAL MIGRATION

At the opposite end of the migratory energetics continuum from income migrants are those animals that complete migration without feeding en route. Perhaps the most celebrated example is the bar‐tailed godwit (*Limosa lapponica*), which completes a ~11,700 km migration from Alaska to New Zealand without interruption (Battley et al., 2012), but this strategy is seen in other shorebirds (Conklin et al., 2017; Gunnarsson & Guðmundsson, 2016; Whitfield et al., 1996), as well as some goose populations (Vissing et al., 2020). Such ‘capital migrants’ rely solely on energy stores built up prior to departure to sustain them throughout the entirety of their journey, minimising the time spent between the departure and destination sites (Lindström et al., 2019). However, non‐stop travel is predicted to be disproportionately expensive because energy stores must themselves be transported until they are required (Piersma, 1987; though see Kvist et al., 2001) and in the meantime the additional mass may inhibit the ability to evade predators (Houston, 1998; Kullberg et al., 1996; van den Hout et al., 2010; Ydenberg et al., 2004). We, thus, expect to see capital migration only when alternatives are infeasible due to, for example, extensive ecologically unsuitable habitat between the departure and destination sites, when there is strong selective pressure on seasonal arrival time or when the distance travelled is so extensive that longer journey times would prohibit other, mutually exclusive activities (e.g. plumage moult, reproduction) being accommodated within the migratory cycle. It should, however, be noted that possible exceptions to these expectations have been outlined (Conklin et al., 2017) and it has been posited that long‐distance (avian) migrants may be capable of torpor (Carpenter & Hixon, 1988; Hiebert, 1993), facultative hypothermia (Carere et al., 2010; Wojciechowski & Pinshow, 2009) or other, unidentified improvements to energetic efficiency (Piersma et al., 2022).

An exception to the expectation that capital migrants transit quickly between their migratory departure point and their destination is provided by one of the largest animal species on the planet, the humpback whale (*Megaptera novaeangliae*). Food availability at the summer feeding grounds is highly seasonal and their foraging method (‘lunge’ or ‘engulfment’ feeding) is energetically profitable only when prey are super‐abundant (Goldbogen et al., 2007). As such, early arrival offers little improvement in the total feeding opportunity for the summer because prey densities are insufficient to reward the effort of foraging. Selection has thus favoured a migration strategy that minimises energy expenditure (i.e. slow swimming speed: Braithwaite et al., 2015) and, indeed, individuals in superior body condition are later to arrive at the breeding grounds (Irvine et al., 2017).

## 4 INTERMEDIATE STRATEGY MIGRATION

**FIGURE 2:**
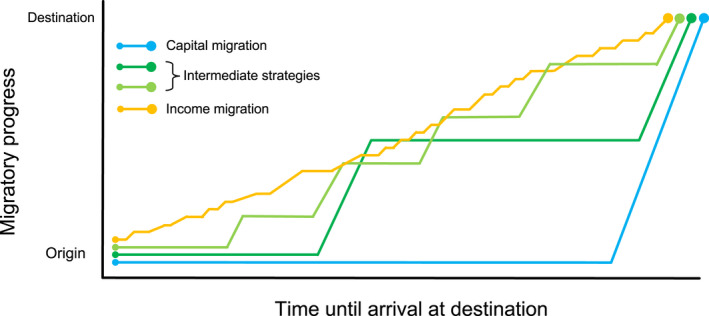
Variation in migratory energetic strategies, from capital migration (blue) to income migration (orange), with intermediate strategies shown in green. Migrants following a capital migration strategy must build sufficient endogenous energy stores to complete the entire journey without refuelling. Intermediate strategy migrants interrupt travel to refuel at one or more stopover sites, typically taking advantage of sites with unusually high resource availability. Refuelling options along their migratory route allow intermediate strategy migrants to depart without sufficient energy stores for the entire migratory journey. However, because energy accumulation during foraging is much slower than is energy consumption during active travel (Hedenström & Alerstam, 1997; Piersma, 1987), intermediate strategy migrants spend much more time overall between their departure and arrival sites than do capital migrants. Income migrants forage opportunistically throughout migration.

The migratory schedules of many migrants lie somewhere between the two extremes of our energetics continuum. These intermediate strategy migrants undertake fasted journeys but do not cover the overall distance in a single effort (Figure 2), making one or more interruptions to the journey to refuel. This is a facet of avian migration biology that is particularly amenable to human observation, and the accessibility of empirical verification perhaps explains the sustained attention that stopover ecology has received from theoreticians, building on Alerstam and Lindström's (1990) influential analytical model. This body of work supports the suggestion that migrating with minimal fuel stores is optimal if the objective is to minimise energy expenditure. However, temporal constraints are ever‐present for an explicitly seasonal lifestyle and each stopover imposes a time penalty due to, for example, deviation from the most direct route to the destination, settling at the new location and time spent foraging and storing fat (Alerstam & Hedenström, 1998). As a result, the increased range capacity that comes with refuelling must be balanced against interruptions to progress. How this is resolved defines the placement of an individual or population along the capital:income continuum and is why theoretical treatment of migration emphasises the importance of considering stopovers (and pre‐departure fat‐loading) to be a part of migration, rather than interruptions to it (Alerstam & Lindström, 1990).

It's worth noting that an individual's position on the energetics continuum need not be the same for each journey within the migration cycle. For example, various shorebird species migrating from Australia to breeding sites in East Asia make a non‐stop flight across the Pacific Ocean of 4400–9000 km but the return journeys to their wintering grounds typically incorporate stopovers on Pacific islands (Zhao et al., 2017). This difference is consistent with stronger selection for early arrival at the breeding grounds, such that a time minimisation strategy is followed (Alerstam & Lindström, 1990), whereas time pressures are eased for the post‐breeding migration and an energy minimisation strategy is more favourable. Conversely, bar‐tailed godwits may fly non‐stop from their Alaskan breeding grounds to non‐breeding sites in New Zealand but when migrating north to breed they make a lengthy detour, stopping on the Yellow Sea coast (Battley et al., 2012). It has been suggested that the prevailing winds of the Pacific Ocean mean that a clockwise circuit of the ocean basin may be more energy efficient when combined with a refuelling stopover in the Yellow Sea (Gill et al., 2014): prevailing winds explain highly indirect oceanic migration routes in seabirds (Felicísimo et al., 2008). We acknowledge that not all migratory interruptions will be motivated by refuelling (Schmaljohann et al., 2022). Migrants may, for example, interrupt active travel to rest. Nursing humpback whales will pause transoceanic migrations in shallow waters en route, which is thought to allow their calves an opportunity to replenish their energy stores through suckling. Mothers do not feed during these stopovers and so we consider them capital migrants, although their calves are functionally income migrants, relying on their mother's milk to fuel both migration and growth (Chittleborough, 1965).

## 5 PHENOTYPIC FLEXIBILITY

Unlike the aerial foragers mentioned earlier, many bird species cannot travel and forage concurrently. Accordingly, avian migrants often adopt capital or intermediate migration strategies, involving distinct periods of feeding and of fasted travel. Performance trade‐offs across activities mean that the phenotype cannot simultaneously be optimised for both travel and feeding, and many avian migrants increase their capacity to endure prolonged flights by reversibly reorienting their phenotype toward flight (and thus away from feeding; Piersma & Lindström, 1997; Piersma, 1998). This migration‐associated ‘phenotypic flexibility’ – a reversible form of phenotypic plasticity (Piersma & Van Gils, 2011) – is seen for morphological, behavioural and physiological traits, and is best exemplified by hyperphagy. Hyperphagy is widely observed in advance of travel and involves changes to all three trait types, with foraging intensity, body mass and enzymatic activity all shifting to support anabolic metabolism, particularly the increased endogenous storage of fat. Fat loads of 40%–45% of total weight occur regularly (hummingbird: Odum & Connell, 1956; shorebirds: Jehl Jr., 1997; Piersma & Gill, 1998) but result not just from the deposition of large quantities of subcutaneous and intraperitoneal fat but also a reduction in the mass of the digestive system, kidneys and liver (Dolnik & Blyumental, 1967; Landys‐Ciannelli et al., 2003; Piersma et al., 1999). This shrinkage of the digestive system does not counterbalance the increase in body weight through fat storage, necessitating an accompanying increase in the size of flight muscles and in aerobic capacity to support them (Marsh, 1981). All these morphological changes are consistent with a trade‐off between flight and feeding capabilities (Piersma, 1998). However, the degree of phenotypic specialisation will depend on the frequency that switches between feeding and locomotion occur. If this is high (income migration) then a compromise will be more strongly favoured than if it is low (i.e. capital end of the continuum), whereupon the costs of phenotypic restructuring are more likely to be outweighed by the greater foraging and travelling efficiencies delivered by temporary phenotypic reorganisation. Within‐species comparison of populations that differ with respect to feeding ecology indicate that the degree of phenotypic flexibility is selected to maximise net metabolic efficiency (van Gils et al., 2005).

While most extensively described in avian migrants, migration‐associated phenotypic flexibility is not limited to birds. For example, hoary bats (*Lasiurus cinereus*) adjust the mass of their digestive system and flight muscles across the migratory cycle. However, in contrast to the avian examples listed above, hoary bats increase the size of their digestive system during migration, thereby maximising refuelling efficiency during the frequent stops en route (McGuire et al., 2013). These bats, then, are an aerial example of income migrants, since the ability to sequester nutrients is upregulated during migration. Western sandpipers (*Calidris mauri*) similarly eschew the patterns seen in many of their congeners, enlarging their digestive organs during migration (Guglielmo & Williams, 2003). Western sandpipers make many short‐stay stopovers (typically of 1–3 days duration: Iverson et al., 1996), compared with the 24‐day stopover of red knots (*Calidris canutus*), which undertake far lengthier movements between stopovers (Baker et al., 2004) and reduce the mass of their digestive system by more than 60% ahead of departure (Piersma et al., 1999). We, thus, see that the form of phenotypic flexibility is particular to the frequency of feeding interruptions during migration: migration at the income end of the continuum favours rapid fuel loading and the phenotype of the migratory phase, in so much as it differs from the sedentary phase, permits faster energy uptake, at least among aerial migrants. Given that a large majority of the overall duration of migration is actually spent foraging (Figure 2; Alerstam & Hedenström, 1998; Hedenström & Alerstam, 1997; Lindström et al., 2019), such a pattern would make sense when migration is acutely time constrained because improvements in foraging efficiency can more easily deliver reductions in migration duration than can increases in movement speed. During the sedentary phases of the migratory cycle, the relative importance of flight performance must be greater so the digestive system is downregulated, which is opposite to the phenotypic flexibility seen in aerial migrants following a capital migration strategy.

In most humpback whale populations, individuals undertake a fasted return migration in which they feed very little, if at all, for 9 months of the year (Chittleborough, 1965; Whitehead & Moore, 1982). We might thus expect them, too, to exhibit phenotypic flexibility within the migration cycle. However, beyond pre‐departure hyperphagia (Boyd, 2004) significant morphological change across the migration cycle has not been reported. It may be that the far lower metabolic rate of baleen whales restricts the extent to which physiological modification can be reversed within an annual cycle. Alternatively, downregulation of digestive tissues may deliver a net loss to the annual energy budget, given that whales have near‐neutral buoyancy and changes in mass therefore deliver marginal reductions in the energetic cost of movement. The major cost of swimming will be derived from drag, and this would be little influenced by reducing internal organ size if the frontal surface area is unchanged. Thus, we see that the mode of travel (i.e. swimming, flying or walking) strongly influences how migratory energetics shapes the extent and form of migration‐associated phenotypic flexibility.

In some migratory insects, distinct phenotypes are expressed (known as ‘phases’) in association with the movement cycle that dedicate the individual to a transient or sedentary lifestyle in adulthood. For example, some North American populations of monarch butterfly (*Danaus plexippus*) spend summer in the continent's northeast and then migrate to wintering sites in central Mexico, a migratory cycle that typically spans between four and six generations. Migratory phases have a lower basal metabolic rate and expend less energy in flight, increasing their flight range compared to sedentary phases (Schroeder et al., 2020). This whole‐generational specialisation does not alleviate the need to resolve life‐history trade‐offs within migratory phases, however, since the multi‐generational aspect of the migration cycle means that all phases must maintain reproductive capacity, which is a competing energy sink. Indeed, among migratory phases there is variation in the extent to which adults' resources are allocated to travel versus reproduction: as per‐generation travel distances increase during the autumn, gonadal development is repressed and expression of sexual behaviours is downregulated, which extends the life span as much as six‐fold (Herman & Tatar, 2001), ensuring that the autumnal deterioration of weather conditions can be avoided by moving southwards sufficiently fast. Concurrently, the lipid content of monarch butterflies increases through the autumn migration (i.e. across successive generations) as travel distances increase (Brower, 1985). Thus, phases are increasingly shifted toward capital migration as autumn progresses. Other insect species exhibit similar, multi‐generational migrations covering thousands of kilometres (e.g. globe skimmer dragonfly, *Pantala flavescens*: Anderson, 2009; painted lady butterfly, *Vanessa cardui*: Stefanescu et al., 2013), but at present, a detailed understanding of migration‐associated plasticity is lacking (May, 2013), and it is difficult to place insects – and other migratory invertebrates – into the context of the energetics continuum owing to the dearth of relevant research. Thus, interspecific comparisons among insects are not currently possible. However, given the patterns observed among different monarch butterfly phases, we posit that seasonal polyphenisms in other insects will show trends of phenotypic specialisation that are consistent with the expectations of the migratory energetics continuum.

## 6 STRATEGIC PLASTICITY

**FIGURE 3:**
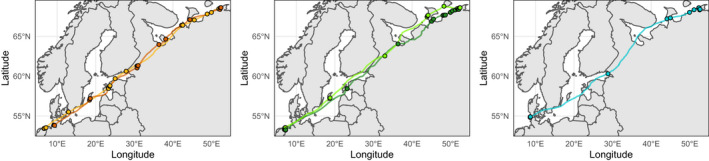
The spring movements of individual barnacle geese (*Branta leucopsis*) from overwintering grounds on the Wadden Sea coast to breeding grounds on the Barents Sea coast. Barnacle geese making their spring migration to the breeding grounds have historically followed an intermediate migratory strategy, stopping to refuel repeatedly in the Baltic and White Seas (left panel), but the increasing availability of agricultural grassland on the Wadden Sea coast has meant that some individuals have shifted toward a capital migration strategy, reaching the Arctic Sea having made only two (centre panel) or even just a single refuelling stop (right panel). Note that refuelling stopovers also vary in duration (Lameris, 2018a), which also shapes the energetic strategy of migration. Plotted using selected individuals (device numbers 6088, 6122, 6131, 6135, 6140) from the archived data of Lameris et al. (2018).

The position of an individual on the capital:income migration continuum may not be fixed, with the possible exception of those occupying the extreme ends of the continuum, which are potentially committed to their migratory schedule by physiological or behavioural inflexibility, or by geographic, temporal or ecological constraints along their route. Strategic plasticity offers resilience against environmental variation, in much the same way as has been envisaged for reproduction (Williams et al., 2017). For example, pink‐footed geese (*Anser brachyrhunchus*) alter their future migration strategy (in terms of stopover refuelling) if they arrive at their breeding grounds in poor condition (Madsen, 2001). Such strategic flexibility also allows migrants to adjust their migration schedule in response to novel foraging opportunities. Barnacle geese (*Branta leucopsis*) overwintering along Europe's North Sea coast have shifted their migratory strategy toward capital migration in response to the improved grazing conditions available on artificially improved agricultural pasture (Fox & Abraham, 2017), which allows them to gain sufficient fat stores to reduce the number of stopovers on the Baltic Sea and White Sea coasts during spring migration (Figure 3; Eichhorn et al., 2009; Lameris et al., 2018). Since fuelling accounts for a large majority of the overall duration of migration (Figure 2; Hedenström & Alerstam, 1997; Lindström et al., 2019), changes to fuelling times are far more impactful on the time taken to reach the destination than increases in the speed of swimming, running or flying. For the barnacle geese, each stopover lasts approximately 1 week (Lameris, Scholten, et al., 2017). Geese that postpone departure from wintering sites for several weeks to continue grazing can thus arrive at the breeding grounds at the same time as individuals persisting with the population's historical multi‐stop migration schedule.

Conversely, reports of humpback whales interrupting travel to feed midway along their migratory route have emerged in various ocean basins in recent years (Danilewicz et al., 2009; Eisenmann et al., 2016; Pinto de sa Alves et al., 2009; Stamation et al., 2007; Stockin & Burgess, 2005). This may reflect a population‐level shift or diversification of migratory strategy in response to decreased abundance of their preferred prey at the traditional feeding grounds (Atkinson et al., 2004; Tulloch et al., 2019), potentially facilitating extended time at breeding sites (Avila et al., 2019). However, the emergence of these observations may alternatively be an artefact of the rapid recovery of baleen whale populations following cessation of intense harvesting (Zerbini et al., 2010).

## 7 PHYLOGENETIC CONSTRAINTS

Some phenotypic features are so fundamental that they cannot be altered by plasticity and major evolutionary changes rarely occur. Examples are the primary mode of locomotion (i.e. swimming, flying or running/walking) and body size, both of which show very strong phylogenetic inertia (i.e. are relatively invariant within lineages) and are impactful determinants of the energetic costs of movement (Blem, 1980; Tucker, 1970). These phylogenetic constraints influence the migratory strategies that populations adopt: for some taxa, long‐distance, fasted travel (i.e. capital and near‐capital migration) will not be a feasible option owing to their evolutionary heritage. For land‐based travel, the relative cost of locomotion is an inverse function of body weight (Tucker, 1970) and migratory lifestyles are predominantly observed among the heavier terrestrial mammals. Even then, the poor energetic economy of walking means that capital migration is feasible only for moderate overland distances (Schmidt‐Nielsen, 1972): the lengthy terrestrial relocations performed by caribou (*Rangifer tarandus*) and various African ungulates migrations are income migrations, with animals foraging as they progress across the landscape. After accounting for body weight, swimming and flying have lower energetic costs than running (Alexander, 2002) and, consistent with this, the migration distances of ungulates are exceeded by more than an order of magnitude by fish, turtles and an array of bird and insect species (Alerstam et al., 2003), many of which are capital migrants, demonstrating the restrictions on migratory strategy that can be imposed by phylogenetic legacy.

## 8 LIFE‐HISTORY IMPLICATIONS

Researchers studying avian migration have long been aware of carryover effects from migration onto reproductive success, mediated by postmigratory residual stores of nutrients (Ankney & MacInnes, 1978; Drent & Daan, 1980; Harrison et al., 2011; Ryder, 1970; Sandberg & Moore, 1996). More particularly, Gudmundsson et al. (1991) noted that breeding migrants following an intermediate migratory strategy may apparently ‘overload’ at their final stopover site, departing with a greater energy reserve than is required to reach the breeding grounds. This energetic surplus is subsequently spent on reproduction. These authors derive a simple formula that predicts that when migrating to a breeding site, additional energy will be stored during refuelling if the net rate of energy gain at the stopover site (after accounting for subsequent transport costs) exceeds the rate of energy gain at the destination, assuming that individuals are selected to complete migration and begin breeding as early as possible. Gudmundsson et al.'s (1991) formula clarifies that we should expect such overloading to be restricted to the final stopover of migration.

Within the Anatidae, a group that has been particularly well studied by migration biologists, across‐species variation in the extent to which pre‐migratory energy stores also fuel reproduction has been linked to body size (Guillemain et al., 2008). This is consistent with ecophysiological constraints: the clutches of smaller species represent a far larger investment relative to adult body weight yet the adults have a proportionally smaller carrying capacity for energy stores (Klaassen et al., 2006; Newton, 2006; Paquette & Ankney, 1999), and it is thus more challenging for them to carry sufficient energy to fuel both migration and breeding (i.e. a combined capital strategy). Nevertheless, even among the passerine birds, which can be orders of magnitude smaller than anatids, arriving at the breeding grounds with higher fat levels is associated with greater reproductive success (Smith & Moore, 2003), perhaps because it allows individuals to begin the early phases of reproduction (e.g. territory establishment, mate selection, nest building) ahead of leaner conspecifics (Kerlinger, 1989; Newton, 2006; Ojanen, 1984; Smith & Moore, 2003), such that the size of individuals' fat stores are not determined – either ecologically or evolutionarily – by the energetic demands of migration alone. Given that migration in these populations can be as indispensable to reproduction as mating or clutch incubation, it may be instructive to treat migration as a facet of reproductive investment, rather than as a distinct life‐history phase. Indeed, juvenile numbers at wintering grounds can be predicted by the average body condition in the preceding late‐winter (Guillemain et al., 2008; Harrison et al., 2013) or early in spring migration (Ebbinge & Spaans, 1995; Madsen, 1995), consistent with a co‐dependency of migration and reproduction on the same energy deposits.

## 9 THE VALUE OF A MIGRATORY ENERGETICS PERSPECTIVE TO CONSERVATION

While human actions have made novel migratory schedules feasible for various species of geese by altering food availability away from the breeding grounds (Fox & Abraham, 2017), the degradation, diminishment or conversion of natural habitats is obliging many migrants to travel greater distances between foraging opportunities (Xu, 2019), such that they must shift towards the capital end of the migratory energetics continuum if they are to survive. Certainly, within migrant populations there is individual‐level variation among migratory behaviours (e.g. timing, route: Franklin et al., 2022; Iverson et al., 1996; Kürten et al., 2022; Zhao, 2016; Verhoeven et al., 2019), including position on the migratory energetic continuum (Hooijmeijer et al., 2014; Figure 3), suggesting they have some capacity to adapt to altered environments by shifting their energetic strategy. However, as Warnock (2010) observed, migrants making a small number of refuelling stops (i.e. near‐capital migrants) tend to be highly faithful to their favoured sites, which are typically characterised by a superabundance of high‐profit food items (Piersma et al., 2021). Such dietary specialisation makes a population vulnerable to extinction if the abundance of their preferred prey declines because they cannot easily switch to alternatives that can be foraged and digested efficiently, or lack alternative sites where their favoured prey is available (Piersma et al., 2021; Piersma & Baker, 2000).

Income migrants, by contrast, rely on the spatial continuity of suitable foraging habitat. For terrestrial species, this has been threatened by the massive changes in land use that have accompanied the emergence of modern human society; the demise of long‐distance migrations by wildebeest (*Connochaetes taurinus*) in Africa and caribou (*Rangifer tarandus*) in North America has been attributed to changes in land use (Manseau et al., 1996; Williamson et al., 1988). This is recognised in calls to create ‘migration corridors’ that protect the passage of animals along viable migration routes by linking up nature reserves and other protected areas. However, those corridors that have been established are often too small to support through‐passage of sizeable populations of large herbivores (which, as described earlier, have high calorific needs), leading to more intense grazing within the protected areas they connect, to compensate for the necessarily accelerated passage through impoverished sections of the corridor (a phenomenon also observed for intermediate strategy migrants in response to the degradation of stopover sites: Liu et al., 2021). This forced shift away from the income end of the energetic continuum is, in the case of African elephants (*Loxodonta africanus*), associated with extensive damage to trees because of the intensification of herbivory in those areas still able to support foraging (Douglas‐Hamilton et al., 2005), thus shifting but not attenuating the pressure on ecosystem function and sustainability.

## 10 IMPACTS OF CLIMATE CHANGE

It is well established that climate warming is progressing fastest at high latitudes (Serreze et al., 2009), such that income and intermediate strategy migrants whose routes cross latitudinal zones are facing a narrowing time window for migration as the arrival of spring becomes increasingly synchronised along migratory routes (Clausen & Clausen, 2013; Lameris, Jochems, et al., 2017). Surfing or hopping over spring's ‘green wave’ (Bischof et al., 2012) will become increasingly difficult as global warming continues, obliging herbivores to alter their migratory strategy or face an increased extinction risk (Møller et al., 2008). For some species that cover modest distances during migration, the necessary strategic change may be readily achievable. The migration of mule deer (*Odocoileus hemionus*), for example, takes 3 weeks, with individuals stopping every 5–7 km, such that only 5% of the time between the beginning and end of migration is spent actively travelling (Sawyer & Kauffman, 2011). Sawyer and Kauffman (2011) speculate that this incremental progress allows mule deer to match their migratory progress to plant phenology and that their migratory journeys could be completed non‐stop in only a few days if necessary. For a moderate sized mammal, a fasted migration of this duration seems feasible. For lengthier migrations, where participants rely on stopover sites to replenish energy stores, the disruption caused by increasing regional synchronisation of spring may be too great a challenge to overcome, since peaks in food availability will no longer be temporally staggered along their migration route and a switch towards capital migration may not be feasible due to environmental or phenotypic constraints. Barnacle geese migrating to the European Arctic miss out stopovers in years when spring is relatively early, suggesting shifts along the migratory energetics continuum can be accommodated via behavioural plasticity, but there are negative impacts on subsequent reproduction (Lameris et al., 2018). Thus, this strategic flexibility may not buffer the population against sustained environmental disruption.

Climate change is also expected to alter marine and atmospheric currents, thereby changing the energetic costs of long‐distance movements through these environments. Given the complexity of these dynamics, and of how migrants' food webs will respond to them, it is probably impossible to make forecasts with any useful degree of certainty (Lennox et al., 2016). However, migrants that travel by swimming or flapping flight are expected to increase energetic expenditure when experiencing currents against their direction of travel and to reduce them when experiencing currents consistent with their intended direction of travel (Hedenström et al., 2002). This means that increased variation in seasonal conditions will make the cost of migration less predictable. Migrants will be selected to buffer themselves against more costly travelling conditions by ‘overloading’: migrating with a greater surplus of energy stores (Weber et al., 1998). Since many avian species appear to depart with near‐maximal fat loads (Jehl Jr., 1997), this may not be achievable via phenotypic flexibility, requiring evolutionary change in skeletal size to increase loading capacity. Such evolutionary change requires a multi‐generational time‐scale so may not occur rapidly enough to keep up with the unprecedented pace of global warming, and if it does it will involve trade‐offs with other vital processes. Thus, migrants at the capital end of the energetics continuum may be more acutely constrained in their ability to accommodate climate change. We must also recognise that for populations close to either end of the continuum, only shifts toward intermediate strategies may be noticeable to biologists and thus reported, thereby leading any literature review to conclude that income and capital migration are expected to decline in response to climate change. Many populations at the extremes may be ‘trapped’ in their particular migratory niche and thus we will see no shift in strategy even if persistence of these populations is severely threatened.

## 11 SUMMARY

We have proposed a typology that highlights the strategic commonalities observed across migrant taxa, ranging from small invertebrates to the largest whales. This framework depicts a continuum of migratory energetic strategies bounded at one end by income migrants, which forage as they travel, such that organisms switch frequently between anabolic and catabolic activities during migration, and at the other by capital migrants, which rely on endogenous energy stores to complete migration without feeding interruptions. Besides clarifying the common selective pressures facing migrants from distantly related taxa and the evolved responses, our organising framework corresponds with theoretical models of migratory strategies, helps us to predict whether and how they may respond to environmental change and provides a vocabulary to unify the distinct specialities within migration research.

## AUTHOR CONTRIBUTIONS

Simon R. Evans conceived the presented framework in discussion with Stuart Bearhop, and this was further developed jointly by both authors. Simon R. Evans led the writing of the manuscript with input from Stuart Bearhop. Both authors contributed intellectual content to drafts and gave their final approval for publication.

## CONFLICT OF INTEREST

The authors have no conflicts of interest.

## Data Availability

Data used to produce Figure 3 were accessed at the online Mendeley Data repository https://data.mendeley.com/datasets/wkv96vcvnj/1 Lameris (2018b).
